# Cognitive inhibition deficit in long COVID-19: An exploratory study

**DOI:** 10.3389/fneur.2023.1125574

**Published:** 2023-04-14

**Authors:** Jacob Saucier, Caroline Jose, Zaynab Beroual, Mohammad Al-Qadi, Simon Chartrand, Eméraldine Libert, Marie-Claire Losier, Kendra Cooling, Gabriel Girouard, Jalila Jbilou, Ludivine Chamard-Witkowski

**Affiliations:** ^1^Faculté de Médecine et des Sciences de la Santé, Université de Sherbrooke, Sherbrooke, QC, Canada; ^2^Center de Formation Médicale du Nouveau-Brunswick, Moncton, NB, Canada; ^3^Vitality Health Network, Dr. Georges-L.-Dumont University Hospital Centre, Moncton, NB, Canada; ^4^School of Psychology, Université de Moncton, Moncton, NB, Canada

**Keywords:** post-COVID syndrome, cognition, inhibition, psychological outcomes, executive syndrome

## Abstract

**Background and objectives:**

An increasing number of research studies point toward the importance and prevalence of long-term neurocognitive symptoms following infection with COVID-19. Our objectives were to capture the prevalence of cognitive impairments from 1 to 16 months post-COVID-19 infection, assess the changes in neuropsychological functions over time, and identify factors that can predict long-term deficits in cognition.

**Methodology:**

A cross-sectional research design was adopted to compare four sub-samples recruited over a 16-month timeframe (1–4, 5–8, 9–12, and 13–16 months). Phone interviews were conducted at least 6 weeks after being infected by COVID-19. Sociodemographic and clinical questionnaires were administered followed by standardized neurocognitive and psychological tests and health questionnaires screening cognitive symptoms, anxiety, depression, fatigue, and autonomy.

**Results:**

Regarding general health questionnaires, 55.2% of the 134 participants had symptoms of psychiatric illness, while 21.6% of patients had moderate-to-severe anxiety or depression. Cognitive efficiency was diminished in 19.4% of our population. Executive dysfunction was screened in 56% of patients, and an impairment of cognitive flexibility and inhibition was revealed in 38.8%. Depression, hospital or intensive care unit (ICU) admission, and the duration of hospital or ICU stay were associated with an inhibition deficit. The duration elapsed from the initial infection, and the neurocognitive assessment was not associated with a decrease in inhibition deficit. The prevalence of cognitive impairments, other than inhibition deficit, tended to decrease during the study period.

**Discussion:**

This study supports the extensive literature on the cognitive and neuropsychiatric sequelae of COVID-19 and highlights long-lasting inhibition deficits, while other cognitive functions seemed to improve over time. The severity of infection could interact as a catalyst in the complex interplay between depression and executive functions. The absence of a relation between inhibition deficits and sociodemographic or medical factors reinforces the need for cognitive screening in all COVID-19 patients. Future research should focus on inhibition deficits longitudinally to assess the progression of this impairment.

## 1. Introduction

Coronavirus epidemics in the past, such as severe acute respiratory syndrome (SARS) and Middle East respiratory syndrome (MERS), had been responsible for long-lasting cognitive impairment, fatigue, mood disturbances, and anxiety. With the arrival of SARS-2 in 2019, the research community anticipated this phenomenon and promptly began investigating the neuropsychological effects of COVID-19 infection (1). Since then, several cohort studies have reported a prevalence of cognitive impairment sometimes exceeding 50% in post-COVID-19 syndrome patients (2–9), defined as the incidence of symptoms at least 12 weeks following the initial COVID-19 infection (10). Specifically, researchers have observed significant deficits in verbal fluency, memory, attention, and executive functions (2, 3, 5–7, 11–13). A recent study evaluating the cognitive functions of post-COVID-19 syndrome patients with subjective cognitive complaints found that attention deficits were the most prevalent cognitive impairment, followed by executive function deficits affecting 61 and 42% of patients, respectively (6). Studies have also reported a high prevalence of neuropsychiatric symptoms including depression, anxiety, fatigue, and sleep disorders in patients following COVID-19 infection (14). Clinical observational studies noted that cognitive dysfunction was exacerbated by anxiety and depressive symptoms, suggesting a possible role of some psychological variables (12). With regard to infection severity, patients requiring intensive care have been shown to have more frequent cognitive impairments and lower scores on neuropsychological tests following acute COVID-19 recovery when compared to patients in a non-ICU setting (15, 16). However, impairments in executive functioning have also been reported by Hennegan et al. to be the most prevalent cognitive outcome in mild-to-moderate COVID-19 survivors (17), which should alert the medical community on the high prevalence of long-term cognitive impact of COVID-19 infection regardless of the severity of the infection symptoms. Mild-to-moderate COVID-19 was measured by Hennegan et al. (17) using the COVEX questionnaire that defines illness severity as mild (“dry cough, headache, nausea/diarrhea, aches and pains, low-grade fever, no need to see a doctor or hospitalization”), moderate [“coughing, high fever (above 100.0 ° Fahrenheit or 37.8°C), chills, feeling that you cannot get out of bed, shortness of breath”], severe (“breathlessness, complications leading to pneumonia”), and critical (“respiratory failure, septic shock, and/or organ dysfunction or failure”) (17).

The orbitofrontal cortex plays a critical role in regulating inappropriate actions, thoughts, and emotions, referred to as cognitive inhibition (18). Increased rumination, negative emotions (19), and anhedonia (20) are also associated with impaired cognitive inhibition. Slower reaction times during the Stroop test’s interference suppression task have been demonstrated in COVID-19 patients when compared to healthy controls (21, 22), correlating with a diminished capacity to suppress inappropriate or irrelevant answers (23).

Several hypotheses are proposed to explain the neurocognitive symptoms of COVID-19, including direct viral invasion, inflammatory alterations, hypoxia, coagulopathy, and vascular endothelial dysfunction (24). The SARS-CoV-2 uses angiotensin-converting enzyme 2 (ACE2) receptors to invade the host cells, leading to a potent inflammatory reaction (25–27). It should be noted that it may invade the olfactory bulbs through the olfactory tract’s ACE2 receptors, causing endothelial cell damage and leading to inflammation, thrombi, and brain damage (28, 29). Similarly, central nervous system (CNS) invasion could be possible through ACE-2 expressing endothelial cells of the blood–brain barrier (BBB) (30–32) and choroid plexus cells *via* the blood–cerebrospinal fluid barrier (BCSFB) (33, 34). Alternatively, neurocognitive symptoms of COVID-19 could occur in the absence of direct viral invasion. The well-documented systemic inflammation could be sufficient to alter BBB permeability and lead to the translocation of inflammatory cytokines and cells into the CNS (35, 36), leading to microglial activation and persistent neuroinflammation (37, 38). Consequently, systemic inflammation leads to decreased monoamines and trophic factors (28), while microglial activation results in excitotoxicity and alterations in synaptic plasticity in addition to neuronal and oligodendrocyte death (37, 38). COVID-19-related neuropsychiatric manifestations are thus hypothesized to be a secondary phenomenon rather than the result of direct brain infection (28). These neuroinflammatory pathways disrupt CNS homeostasis and are closely linked to cognitive and neuropsychiatric symptoms (26). In accordance, Mazza et al. measured inflammatory markers during COVID-19 infection using the systemic immune-inflammation index (SII), a method of quantifying systemic inflammation. The SII score was found to predict depressive symptoms and neurocognitive dysfunction at 3 months follow-up (9). Finally, brain stem involvement may explain persistent autonomic abnormalities and anxiety (28).

Despite an increasing number of studies reporting neurological deficits in non-hospitalized patients during long-term COVID-19 follow-ups, their definition of the long term can vary substantially from 6 weeks to 6 months (6, 8, 17), with some rare studies with data from 1 year and beyond the initial infection (39–42), explaining the lack of a clear long-term cognitive profiling of COVID-19 survivors. In order to improve our comprehension of the evolution pattern of cognitive profile in those patients, we used a cross-sectional design in which participants were recruited from 6 weeks to 16 months after their COVID-19 infection and assessed for cognitive impairments.

The objectives of the study were to capture the prevalence of cognitive impairments in post-COVID-19 infection, evaluate the changes in neurocognitive functions over time, and identify factors that can predict long-term deficits in cognition.

## 2. Materials and methods

We conducted a cross-sectional research study. The population was sampled randomly over a 16-month period. Data were collected only once from each participant. For evaluation of changes in neurocognitive profiles through time, the sample was divided into two groups with the proxy temporal strata of “1–6 and 7–16 months between acute COVID-19 symptoms and neuropsychological assessment.”

Participants were recruited *via* the University Health Center (UHC) of the Dr-Georges-L.-Dumont Biobank. Participants gave consent at the time of diagnosis of a COVID-19 infection to be contacted for participation in future studies. Participants who had given consent were contacted by the coordinator of the Biobank to assess whether they would like to participate in our study. Only participants who met the inclusion criteria and had none of the exclusion criteria were contacted. Inclusion criteria were as follows: patients (1) being at least 19 years of age, (2) having a positive COVID-19 test made by molecular detection (PCR test, SARS-CoV-2; RCP/TAAN) at least 6 weeks before study enrollment, (3) being able to communicate verbally in English or in French, and (4) being available for a 60-min interview. Exclusion criteria were as follows: patients (1) having a current diagnosis of a major cognitive disorder or dementia and (2) having untreated hearing loss. If participants agreed to participate in the study, they were then contacted by a research assistant who explained the study details and obtained verbal consent to participate in the study.

Phone interviews were conducted by four medical students that had previously benefited from multiple training sessions on the administration of the different questionnaires and the neurocognitive tests to assure standardization of data collection. The tests chosen were aimed at evaluating cognitive efficiency, orientation, attention, immediate and short-term memory, processing speed, and executive functions through mental flexibility comprising inhibition, psychological distress, levels of autonomy, and fatigue. All the neurocognitive scales and the psychological, instrumental, and fatigue questionnaires were validated tools, normed to the general population, and available in English and French (43–50). As our population is bilingual, participants were offered to be interviewed in their preferred language.

The phone interviews were structured as follows:

- Patients were explained the different steps of the interview. Patients were asked to be in a quiet place without any distractions.
- Patients were then asked general questions about their sociodemographic information, past medical history, as well as a history of COVID-19 infection.
- Afterward, the Patient Health Questionnaire (PHQ-9) (43), the Generalized Anxiety Disorder 7-item scale (GAD-7) (44), the Instrumental Activities of Daily Living (IADL) (45), and the Brief Fatigue Inventory (BFI) (46) were administered.
- Patients were allowed a 10-min break to minimize the impact of mental fatigue on subsequent neurocognitive tests.
- Finally, the administration of the neurocognitive tests: Modified Telephone Interview of Cognitive Impairment (TICSm) (47), Oral Trail Making Test A and B (O-TMT A and B) (48), and the Hayling and Brixton test (49).

The Patient Health Questionnaire-9 (PHQ-9) screens depression through nine questions that correspond to the DSM-IV’s (51) criteria for a major depressive disorder, ranking each symptom’s frequency from never (score of 0) to nearly every day (score of 3). The sum of individual symptoms’ scores allows the assessment of the overall severity of the patient’s depression as follows: minimal depression (0–4 points), mild depression (5–9 points), moderate depression (10–14), moderately severe depression (15–19), and severe depression (20–27) (43).

The Generalized Anxiety Disorder-7 (GAD-7) screens anxiety through seven questions that correspond to the DSM-IV’s criteria for generalized anxiety disorder, ranking each symptom’s frequency from never (score of 0) to nearly every day (score of 3). The sum of individual symptoms’ scores allows the assessment of the overall patient’s anxiety as follows: minimal anxiety (0–4 points), mild anxiety (5–9 points), moderate anxiety (10–14), and severe anxiety (15–21) (44).

The Instrumental Activities of Daily Living (IADL) scale evaluates participants’ level of autonomy through the ability to fulfill eight categories of activities of daily living: using a telephone, shopping, food preparation, housekeeping, doing the laundry, mode of transportation, being responsible for own medication, and handling finances (45). Each category of activities is dichotomized in the scores of 1 or 0, depending on the participant’s self-reported ability or disability to fulfill them, respectively. A score of 8 represented total autonomy, a score from 1 to 7 represented a partial loss of autonomy, and 0 represented a total loss of autonomy.

The Brief Fatigue Inventory (BFI) consists of 10 components (46). The first question inquires as to whether the individuals have experienced fatigue in the last 7 days. In total, three items evaluate fatigue magnitude where subjects score their usual level of fatigue, the current level of fatigue, and the worse level of fatigue based on a 0–10 scale of increasing severity, from “no fatigue” to “as bad as you can imagine” fatigue. Interference with daily activities and interpersonal relations is evaluated by six items, using a scale from 0 (does not interfere) to 10 (completely interferes). We calculated the average severity of fatigue and fatigue interference by calculating the sum of individual item severity divided by the number of evaluated items. A score of 0 corresponded to no fatigue, a score from 1 to 3 represented mild fatigue, a score of 4–7 represented moderate fatigue, and a score of 8–10 represented severe fatigue.

The Telephone Interview for Cognitive Status-Modified (TICSm) from the Mayo Clinic is a validated tool for the screening of mild cognitive impairment and dementia, evaluating cognitive efficiency (47). The TICSm correlates highly with the mini-mental state examination (MMSE) (52). This tool also evaluates orientation in time and space, concentration, immediate and short-term memory, calculation, semantic memory, and repetition. It includes the following components: (1) name, date, age, and phone number (maximum score of nine points); (2) counting backward (maximum score of two points); (3) first, a 10-word list learning exercise and then a delayed recall of that word list (maximum score of 10 points each); (4) subtraction (maximum score of five points); (5) responsive naming (maximum score of four points); (6) repetition (maximum score of two points); (7) current president and vice president (maximum score of four points); (8) finger tapping (maximum score of two points); and (9) word opposites (maximum score of two points) (47). The items add up to a total of 50 points. The standardized test classifies the severity of cognitive impairment as follows: “normal, equal, or superior to 33”; “ambiguous, 26–32”; “mildly impaired, 21–25”; and “moderately to severely impaired, inferior, or equal to 20.” We derived a dichotomic variable of cognitive ability, with a cutoff value of 33 points. Therefore, a score equal to or less than 32 points was defined as an abnormal cognitive ability, and a score of more than 33 as a normal cognitive ability. This dichotomization pools ambiguous assessments and mild-to-severe cognitive impairments in the “abnormal” class.

The Oral Trail Making Test A (O-TMT-A) assesses attention and processing speed, whereas the Oral Trail Making Test B (O-TMT-B) is regarded as an executive task and assesses cognitive flexibility, described as the ability to “switch” between mental tasks. In the O-TMT-A task, the examiner instructs the participant to count from 1 to 25 as fast as possible. During the O-TMT-B task, the patient is instructed to count while alternating between the number and the corresponding letter (1-A-2-B, etc.) as fast as possible until the examiner instructs to stop when reaching number 13. The examiner times the subject with a chronometer during both trials. For both tests, an abnormal score corresponds to the 9th percentile of task completion rapidity, which is weighted against the patient’s age group (48).

The Hayling and Brixton Test measures cognitive inhibition. It consists of 30 sentences with the final word missing. In the first section, the test conditions automatisms by having the participant rapidly complete the sentence with an associated word. In the second section, the participant must inhibit this automatism and complete the sentence with a word that has no relation to the sentence’s meaning. The administration takes approximately 5 min. We used the commonly used English version of the test (49) and a French adaptation that has been validated by Bayard et al. (50). The English version’s scores are ranked out of 10, and we classified the four poorest scores (impaired, abnormal, poor, and low average) as abnormal cognitive inhibition and the six best scores as normal cognitive inhibition. The French version is ranked out of 5, and we classified the two poorest scores “pathologic and limit” as abnormal and the others as normal. The use of dichotomic variables interpretation was implemented to enable comparability between the French and English versions.

This study was reviewed and approved by the Vitalité Health Network’s Research Ethics Committee on 18 November 2020 (reference # 10151) and by the Research Ethics Committee of Université de Moncton on 21 March 2021 (Reference # 2021-047).

### 2.1. Statistical analyses

To describe the characteristics of the sample, continuous variables were presented as medians (interquartile range) or means (standard deviation). Binary and categorical variables were presented as counts and percentages. To know whether the length between the start of the infection symptoms and the cognitive evaluation was higher in the group of participants scoring in the normal range compared to the group scoring in the abnormal range of the cognitive tests (TICS-m, O-TMT-A, O-TMT-B, or Hayling), we conducted one-way Mann–Whitney *U*-tests. To further compare the characteristics of the participants according to their scoring on the Hayling and Brixton test or TICS-m test (normal vs. abnormal), as well as according to the time of assessment (early vs. late), χ^2^ tests were used to identify the difference in the proportion across categories, while for normally distributed continuous data, Student’s *t-*tests were used to test differences between groups or Mann–Whitney *U*-tests for non-normally distributed data. We did not conduct multiple testing corrections for statistical comparison for two main reasons: One is that our multiple tests were done on a single data set using different statistical procedures (χ^2^, Student’s *t-*test, and Mann–Whitney *U*-tests). The various procedures are based on different statistical models, so a Bonferroni correction or similar approach may result in conflicting conclusions from the same data, and second, as the context of the study is exploratory, we wish not to miss a possible effect worthy of further study, and therefore, a correction would be inappropriate (53).

All analyses were conducted using the Jamovi (version 1.6) statistical platform (54).

## 3. Results

### 3.1. Study sample

**Figure 1:**
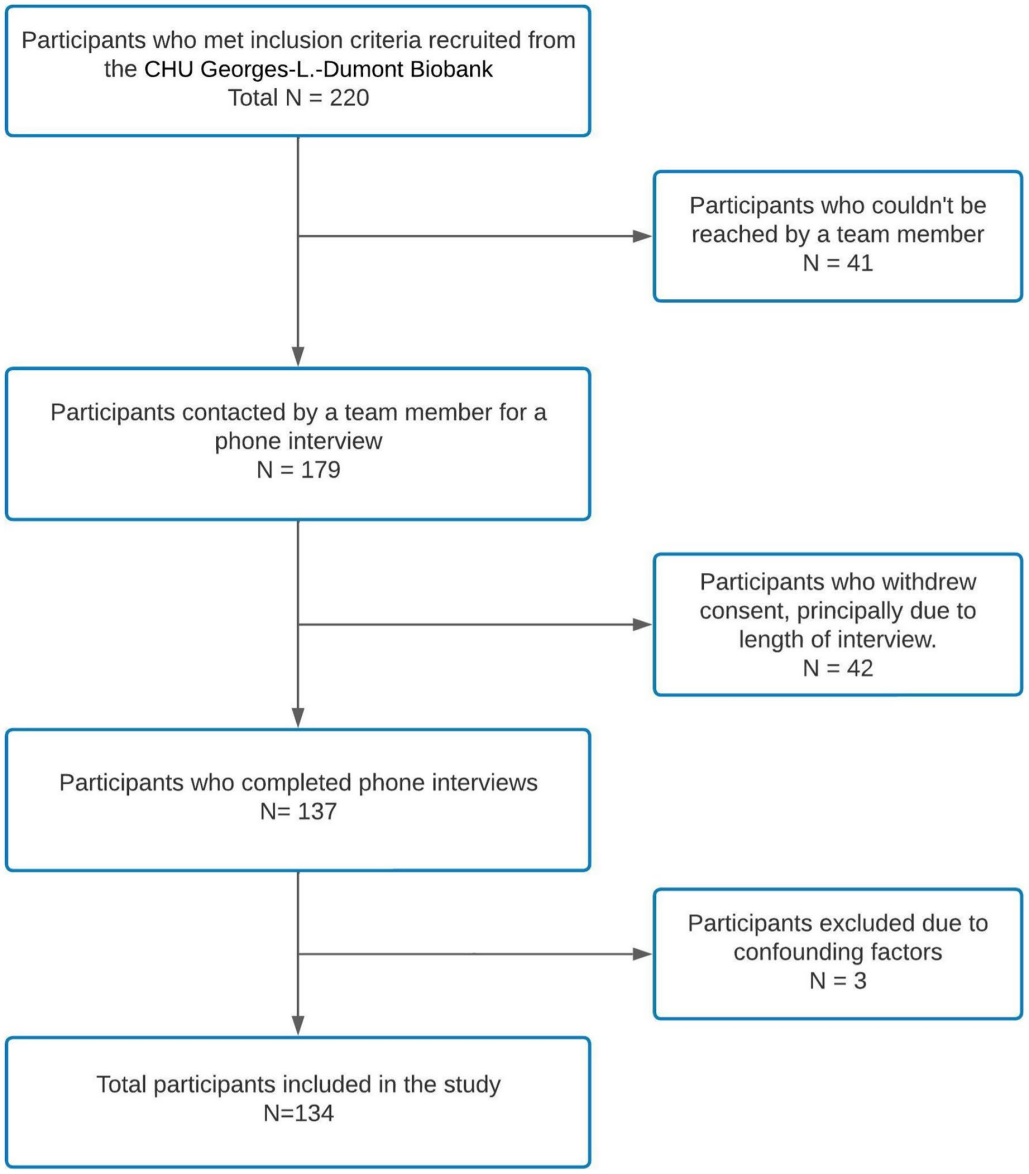
Patient recruitment and inclusion flowchart.

The COVID-19 database from UHC Dr-Georges-L.-Dumont Biobank listed 220 patients who gave consent to be contacted for further research studies and those who met our inclusion criteria. Between September 2020 and August 2021, 179 of the 220 participants answered the phone call offering them to participate in our study. Of the 179 participants who could be reached, a total of 137 participants consented to participate and were included (response rate, 77%). During data analysis, three participants were excluded due to missing data in the cognitive assessment. One participant had to end the call to care for a familial emergency, and team members stopped the cognitive assessment of two participants because of evident fatigue. A total of 134 participants’ data were analyzed (see Figure 1 for the recruitment flowchart).

### 3.2. Description of the participants

**Figure 2:**
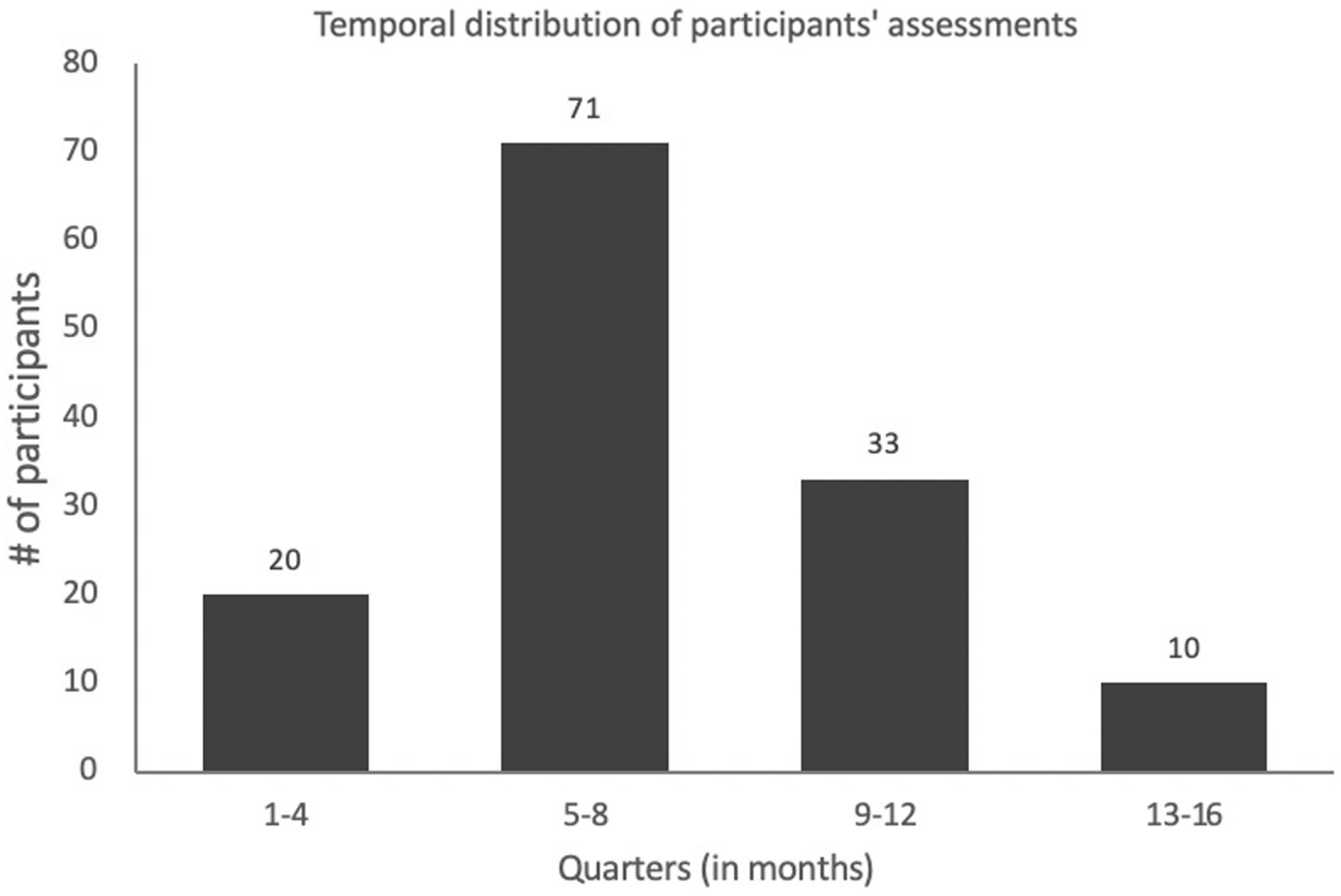
Temporal distribution of the participant’s neuropsychological assessments. 20 patients had their assessment 1-4 months, 71 between 5-8 months, 33 between 9-12 months and 10 patients between 13-16 months following their respective COVID-19 infection onset.

The average duration between the acute COVID infection-related symptoms and the neuropsychological assessment was of 7.7 months (ranging from 1.3 to 16.6 months). The distribution of participants during the study period is depicted in Figure 2.

Of the 134 patients included in our study, the mean age was 50.0 ± 14.5 years and 55% were women (Table 1). In our cohort, 72.4% received post-secondary education. Regarding their past medical history, 62.7% had a body mass index of more than 27, 12.7% had diabetes, 22.4% had hypercholesterolemia, and 26.9% had hypertension. When asked about their past mental health history, 22.4% of participants self-reported past anxiety, while 21.6% reported a previous history of depression. During the acute phase of their COVID-19 infection, the most frequent symptoms were fatigue (77.7%), followed by headache (65.7%) and cough (61.2%). They had an average of two different symptoms during the acute phase of COVID-19 infection, and the symptoms lasted for an average of 44.2 days (95% CI: 32.3–56.1). Hospitalization was reported by 9% of patients with an average hospitalization stay of 1 day (95% CI: 0.3–1.7). Intensive care unit admission was reported in 4.5% of patients, and they stayed in ICU for 0.6 days on average (95% CI: 0.02–1.2).

**Table 1:** Characteristics of the study sample.

	Total population (*n* = 134)
**Demographic characteristics**
Age, in years, mean (SD)	50.0 (14.5)
Sex, female *n* (%)	76 (55)
**Education**
College or technical Diploma, *n* (%)	50 (37)
Undergraduate Diploma, *n* (%)	37 (27.6)
Graduate University diploma, *n* (%)	10 (7.5)
**Past medical history**
Cardiovascular disease risk factors	
High blood pressure, *n* (%)	36 (26.9)
BMI >27, *n* (%)	84 (62.7)
Hypercholesterolemia, *n* (%)	30 (22.4)
Diabetes, *n* (%)	17 (12.7)
**Psychiatric illness history**
Previous anxiety (self-reported), *n* (%)	30 (22.4)
Previous depression (self-reported), *n* (%)	29 (21.6)
**COVID-19 infection history**
**COVID symptoms**
Cough, *n* (%)	82 (61.2)
Fever, *n* (%)	64 (47.8)
Breathing difficulty, *n* (%)	64 (47.8)
Feverish symptoms, *n* (%)	50 (37.3)
Chills, *n* (%)	62 (46.3)
Tired, *n* (%)	104 (77.6)
Taste/smell (hyposmia), *n* (%)	72 (53.7)
Headache, *n* (%)	88 (65.7)
Gastrointestinal issues, *n* (%)	54 (40.3)
Pain, *n* (%)	56 (41.8)
Severe discomfort, *n* (%)	38 (28.4)
Number of symptoms experienced, mean (SD)	2 (1.5)
Symptoms’ duration (days), mean (min-max)	44.0 (0–354)
**Hospitalization**
Hospitalized, *n* (%)	12 (9.0)
Days hospitalized, mean (95% CI)	0.9 (0.3 to 1.7)
ICU admission, *n* (%)	6 (4.5)
Days in ICU, mean (95% CI)	0.6 (0.02 to 1.2)

### 3.3. Prevalence and severity of cognitive impairment in post-acute COVID-19 population

Standardized psychological, fatigue, and instrumental tests were used to evaluate anxiety (GAD-7), depression (PQH-9), autonomy (IADL), and fatigue (BFI) post-COVID-19 infection. Overall, 55.2% of participants had symptoms of psychiatric illness (Table 2). Specifically, the GAD-7 questionnaire screened anxiety of mild severity in 20.9% of patients, moderate severity in 7.5% of participants, and severe anxiety in 5.2%. Regarding depression, the PHQ-9 questionnaire revealed mild symptoms of depression in 20.9%, moderate depression in 11.9%, moderately severe depression in 3.7%, and severe depression in 3.0% of study participants. Mild fatigue was observed in 35.8% of patients, moderate fatigue in 32.8%, and severe fatigue in 2.2% of participants. In total, 70.9% of the study’s population reported mild-to-severe fatigue following their COVID-19 infection. Less than 5% of patients reported a partial loss of autonomy upon completion of the IADL.

**Table 2:** Post-COVID-19 neuropsychological assessment.

Psychological, fatigue, and autonomy	
**GAD-7 (anxiety)**
Absent, *n* (%)	89 (66.4)
Mild, *n* (%)	28 (20.9)
Moderate, *n* (%)	10 (7.5)
Severe, *n* (%)	7 (5.2)
**PHQ-9 (depression)**
Absent, *n* (%)	81 (60.4)
Mild, *n* (%)	28 (20.9)
Moderate, *n* (%)	16 (11.9)
Moderately severe, *n* (%)	5 (3.7)
Severe, *n* (%)	4 (3.0)
**Brief fatigue index**
Absence, *n* (%)	37 (27.6)
Mild, *n* (%)	48 (35.8)
Moderate, *n* (%)	44 (32.8)
Severe, *n* (%)	3 (2.2)
IADL partially autonomous, *n* (%)	6 (4.5)
Mild psychiatric sequelae^1^, *n* (%)	45 (33.6)
Moderate or severe psychiatric sequelae^2^, *n* (%)	29 (21.6)
Psychiatric symptoms^3^, *n* (%)	74 (55.2)
**Neurocognitive functions**
Abnormal TICS-m, *n* (%)	26 (19.4)
Abnormal Hayling and Brixton, *n* (%)	52 (38.8)
Abnormal O-TMT-A, *n* (%)	12 (9.0)
Abnormal O-TMT-B, *n* (%)	12 (9.0)
Abnormal O-TMT A or B, *n* (%)	18 (13.4)
Cognitive impairment,^4^ *n* (%)	78 (58.2)
Executive dysfunction,^5^ *n* (%)	75 (56.0)
Time between COVID and interview, months, mean (min-max)	7.7 (1.3–16.5)

^1^Represents mild anxiety or mild depression.^2^Represents moderate-to-severe anxiety or moderate-to-severe depression.^3^Represents mild, moderate and severe psychiatric sequelae.^4^Represents an abnormal result on at least one of the following tests: Hayling and Brixton, TMT-A, or TMT-B, or an ambiguous or impaired score on the TICS-m.^5^Represents an abnormal result on at least one of the following tests: Hayling and Brixton, TMT-A, or TMT-B.

The neurocognitive assessment revealed deficits in various cognitive functions. With regard to the TICS-m, 19.4% of the participants showed a global impairment of cognitive efficiency. A deficit in cognitive flexibility and inhibition was revealed in 38.8% of patients by an abnormal Hayling and Brixton test result. With regard to the Oral Trail Making Test, 9% of the study’s population had an abnormal score on the O-TMT-A and 9% on the O-TMT-B, although they were not necessarily the same participants having abnormal scores on both tests. Overall, 58.2% of participants showed an abnormal test result on at least one neurocognitive scale.

The scope of cognitive impairment was assessed by the cumulative number of abnormal tests. Mild impairment was defined by only one of the tests being abnormal, moderate impairment by two abnormal tests, moderately severe by three abnormal tests, and severe by more than three abnormal tests. Mild impairment was found in 39.6% of the sample, moderate impairment in 17.2%, and moderately severe in 1.5%. No participant had severe cognitive impairment based on our definition in our sample (Table 3).

**Table 3:** The severity of cognitive impairment based on the cumulative number of abnormal tests.

	Total	Hayling and Brixton	TICSm	O-TMT-A	O-TMT-B
Not impaired, *n* (%)	57 (41.8)	0	0	0	0
Mild (One abnormal test), *n* (%)	53 (39.6)	32 (60,4)	14 (26.4)	3 (5.7)	4 (7.4)
Moderate (Two abnormal tests), *n* (%)	23 (17.2)	19 (82,6)	11 (47.8)	9 (39.1)	6 (26.1)
Moderately severe (Three abnormal tests), *n* (%)	1 (1.5)	1 (100)	1 (100)	0	1 (100)
Severe (Four or five abnormal tests), *n* (%)	0	0	0	0	0

The distribution of specific cognitive impairments varied in our sample. In our population, when a single test was abnormal (mildly impaired), it was preferentially the Hayling and Brixton test (60.4%) followed by the TICS-m (26.4%). When the two tests were abnormal (moderately impaired), the Hayling and Brixton test was the more prevalent (82.6%), followed by the TICS-m (47.8%), the O-TMT-A (39.1%), and the O-TMT-B (26.1%). Cognitive flexibility and inhibition were the predominant cognitive impairments in all severity levels, followed by cognitive efficiency.

### 3.4. Cognitive profile comparison between early and late assessment groups in post-acute COVID-19 syndrome

To assess the potential differences in the cognitive profile of post-acute COVID-19 syndrome with time, we broke down the sample into four groups representing different increments in lengths between the first COVID-19-related symptoms and the neuropsychological assessment (four quarters: 1–4, 5–8, 9–12, and 13–16 months). Overall, 20 participants had their assessment in the first quarter post-acute COVID-19, 71 in the second quarter, 33 in the third, and 10 in the last quarter.

**Figure 3:**
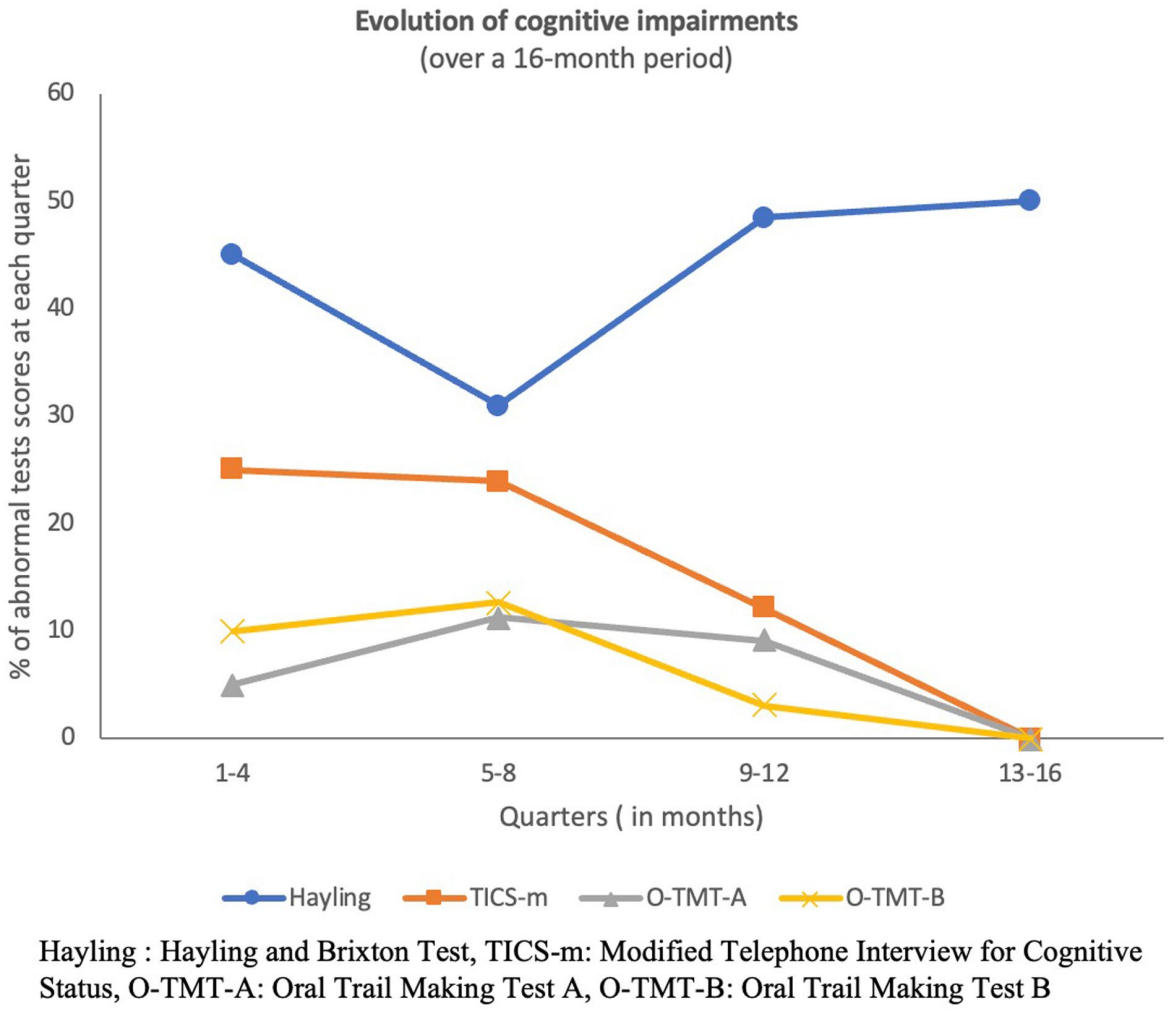
Cross-sectional evolution of cognitive impairments throughout the study period.

The prevalence of abnormal scores in the four cognitive tests for each of the four sub-samples is presented in Figure 3. The prevalence of abnormal scores decreased in three of the four tests and ended at zero in the last 4 months of the study period for O-TMT-B, O-TMT-A, and TICS-m. However, abnormal Hayling and Brixton scores remained high with a prevalence of 50% even up to 16 months post-infection.

Because of the low number of participants in some of the quarters limiting statistical power for subsequent analyses, we stratified the sample into two groups: one having been assessed from 1 to 6 months post-acute COVID-19 infection (early assessment) and one assessed from 7 to 16 months post-acute COVID-19 infection (late assessment). We had 86 participants in the early assessment group and 48 in the late assessment group. In terms of sociodemographic characteristics, the two groups only differed by the preferred language (*p* ≤ 0.001) and education level (*p* = 0.010). The late assessment group comprised more anglophone participants and more post-secondary education levels, and in addition, a greater number of hospitalized patients (*p* = 0.020). Abnormal scores in O-TMT-A and Hayling and Brixton tests, respectively, did not differ between groups, but the prevalence of cognitive efficiency impairments (TICS-m) and O-TMT-B was significantly higher in the early assessment group (*p* = 0.020 and *p* = 0.036, respectively).

The distribution of cognitive impairment severity levels in the early and late assessment groups is shown in Table 4. There was no difference in the prevalence of any severity level of impairment between the two groups. The only patient having moderately severe impairment was in the early assessment group.

**Table 4:** The prevalence of the severity levels of cognitive impairment in the early and late assessment groups.

	Early assessment group (*n* = 86)	Late assessment group (*n* = 48)	*p* value
Not impaired, *n* (%)	33 (38.4)	24 (50.0)	0.140
Mild (One abnormal test), *n* (%)	37 (43.0)	16 (33.3)	0.098
Moderate (Two abnormal tests), *n* (%)	15 (17.4)	8 (16.7)	0.166
Moderately severe (Three abnormal tests), *n* (%)	1 (1.2)	0 (0.0)	n/a
Severe (Four or five abnormal tests), *n* (%)	0 (0.0)	0 (0.0)	n/a

n/a, not applicable.

### 3.5. Risk factors of cognitive flexibility and inhibition and for cognitive efficiency impairments

We tested whether more symptoms of COVID infection or a longer duration of COVID symptoms were associated with abnormal scores in the four cognitive tests and found no significant association with any of the tests.

Given the low prevalence of O-TMT-A and O-TMT-B abnormal scores, we focused the analyses of cognitive impairment risk factors on the TICS-m and Hayling and Brixton scales. Because the prevalence of cognitive efficiency impairments (TICS-m) and cognitive flexibility and inhibition (Hayling and Brixton) showed little overlap, we decided to further evaluate the factors associated with those two cognitive functions separately. We first compared all of the variables between the normal and abnormal TICS-m score groups and between the normal and abnormal Hayling and Brixton score groups with univariate analyses.

Regarding TICS-m groups (Table 5), more participants in the abnormal TICS-m group had symptoms of anxiety at the time of the assessment with the GAD-7 scale (*p* = 0.004) and symptoms of depression when assessed with the PQH-9 scale (*p* = 0.044). Participants in the normal TICS-m group had higher education levels than participants in the abnormal group (*p* = 0.028). There was no significant difference between the groups in terms of their medical history.

**Table 5:** Comparison of normal and pathologic m-TICS test groups.

	Normal (*n* = 108)	Abnormal (*n* = 26)	*p* value
**Sociodemographic characteristics**
Female, *n* (%)	61 (56.5)	15 (57.7)	0.914
Age, median (IQR)	49.6 (15.0)	51.8 (12.7)	0.719
English Preferred language, *n* (%)	61 (56.5)	15 (57.7)	0.911
Income, mean (SD)	57,525 (34,883)	59,200 (32,554)	0.739
College or university diploma, *n* (%)	82 (75.9)	14 (53.8)	<0.05^**^
Duration between first COVID symptoms and assessment in days, mean (SD)	242 (104)	197 (80)	<0.10^*^
**Past medical history**
Body mass index >27, *n* (%)	70 (65.4)	14 (53.8)	0.272
Anxiety, *n* (%)	23 (21.3)	7 (26.9)	0.537
Depression, *n* (%)	23 (21.3)	6 (23.1)	0.843
Neurological disorders, *n* (%)	1 (1.2)	0 (0.0)	n/a
High blood pressure, *n* (%)	29 (26.9)	7 (26.9)	0.994
High cholesterol, *n* (%)	25 (23.1)	5 (19.2)	0.667
Diabetes, *n* (%)	14 (13.1)	3 (11.5)	0.832
Cerebrovascular accident, *n* (%)	0 (0.0)	2 (7.7)	n/a
Concussion, *n* (%)	10 (9.3)	4 (15.4)	0.359
COPD, *n* (%)	0 (0.0)	0 (0.0)	n/a
**COVID-19 infection symptoms**
Cough, *n* (%)	64 (59.3)	16 (61.5)	0.832
Breathing difficulties, *n* (%)	51 (47.2)	12 (46.2)	0.922
Fever-like symptoms, *n* (%)	69 (63.9)	19 (73.1)	0.376
Fatigue, *n* (%)	79 (73.1)	21 (80.8)	0.423
Musculoskeletal pain, *n* (%)	61 (56.5)	16 (61.5)	0.640
Headache, *n* (%)	64 (59.3)	20 (76.9)	<0.10^*^
Gastrointestinal symptoms, *n* (%)	41 (38.0)	11 (42.3)	0.683
Taste–smell problem, *n* (%)	55 (50.9)	14 (53.8)	0.789
Duration of symptoms, days, and mean (SD)	42.4 (70.5)	50.8 (69.6)	0.839
Number of symptoms, mean (SD)	4.8 (2.8)	5.2 (2.1)	0.584
Severe discomfort, *n* (%)	30 (27.8)	6 (23.1)	0.627
Hospitalization, *n* (%)	11 (10.2)	1 (3.8)	0.310
Duration of hospitalization in days, mean (SD)	1.2 (4.6)	0.2 (0.9)	0.307
Admission in ICU, *n* (%)	5 (4.6)	1 (3.8)	0.862
Duration of ICU admission in days, mean (SD)	0.73 (3.9)	0.19 (0.98)	0.844
**Post-COVID-19 neuropsychological assessment**
IADL partially autonomous, *n* (%)	6 (5.6)	0 (0.0)	0.219
BFI severe, *n* (%)	3 (2.9)	0 (0.0)	<0.01^*^
PHQ-9 mild to severe, *n* (%)	38 (35.2)	15 (57.7)	<0.05^**^
GAD-7 mild to severe, *n* (%)	30 (27.8)	15 (57.7)	<0.01^***^
Abnormal Hayling, *n* (%)	41 (38.0)	11 (42.3)	0.683
Abnormal O-TMT-A, *n* (%)	12 (11.1)	0 (0.0)	<0.10^*^
Abnormal O-TMT-B, *n* (%)	10 (9.3)	2 (7.7)	0.802

SD, standard deviation; IQR, interquartile range; COPD, chronic pulmonary obstructive disorder; ICU, intensive care unit; GAD-7, generalized anxiety disorder-7; PHQ-9, patient health questionnaire-9; IADL, instrumental activities of daily living; BFI, brief fatigue index; TICS-m, modified telephone interview for cognitive status; O-TMT-A, oral trail making test A; and O-TMT-B, oral trail making test B.

A χ^2^ test was used to identify differences in proportions across categories. For normally distributed continuous data, a Student t-test was used to test differences between groups or by the Mann–Whitney U-test for non-normally distributed data. Bold characters indicate significant differences (*p* ≤ 0.05). *p* value < 0.1 (^*^), *p* value < 0.05 (^**^), and *p* value < 0.01(^***^).

Regarding the Hayling and Brixton groups (Table 6), there was neither difference in their medical history, and in any COVID-19-related symptoms, nor there were differences in sociodemographic characteristics between the two groups. Looking at the neuropsychological tests battery, a significant difference between our two groups was found only for depression symptoms as screened by PQH-9 (*p* = 0.046). *S*ignificant differences were also found for hospital admission, length of hospital stay, intensive care unit (ICU) admission, and length of ICU stay (*p* < 0.05). When those significant variables were included in a multivariate regression model, only depression remained significant (*p* = 0.047). More specifically, severe depression was associated with abnormal scoring on the Hayling and Brixton test (*p* = 0.021), while moderate depression was not significant.

**Table 6:** Comparison of normal and pathologic Hayling and Brixton’s test groups.

	Normal (*n* = 82)	Abnormal (*n* = 52)	*p* value
**Sociodemographic characteristics**
Female, *n* (%)	45 (54,9)	31 (59.6)	0.590
Age, median (IQR)	53 (23.8)	56 (22.5)	0.955
English preferred language, *n* (%)	46 (56.1)	30 (57.7)	0.856
Income, mean (SD)	54,367 (28,297)	63,723 (41,526)	0.530
College or university diploma, *n* (%)	56 (68.3)	40 (76.9)	0.282
Duration between first COVID symptoms and assessment in days, mean (SD)	226 (10.3)	245 (15.4)	0.536
**Past medical history**
Body mass index >27, *n* (%)	54 (65.9)	30 (57.7)	0.295
Anxiety, *n* (%)	20 (24.4)	10 (19.2)	0.485
Depression, *n* (%)	20 (24.4)	9 (17.3)	0.332
Neurological disorders, *n* (%)	1 (1.2)	0 (0.0)	n/a
High blood pressure, *n* (%)	23 (28.0)	13 (25)	0.698
High cholesterol, *n* (%)	21 (25.6)	9 (17.3)	0.261
Diabetes, *n* (%)	7 (8.5)	10 (19.2)	0.074
Cerebrovascular accident, *n* (%)	1 (1.2)	1 (1.9)	n/a
Concussion, *n* (%)	9 (11.0)	5 (9.6)	0.802
COPD, *n* (%)	0 (0)	0 (0)	n/a
**COVID-19 infection Symptoms**
Cough, *n* (%)	50 (61.0)	30 (57.7)	0.706
Breathing difficulties, *n* (%)	37 (45.1)	26 (50.0)	0.581
Fever-like symptoms, *n* (%)	49 (59.8)	39 (75.0)	<0.10^*^
Fatigue, *n* (%)	61 (74.4)	39 (75.0)	0.931
Musculoskeletal pain, *n* (%)	47 (57.3)	30 (57.7)	0.966
Headache, *n* (%)	49 (59.8)	35 (67.3)	0.378
Gastrointestinal symptoms, *n* (%)	31 (37.8)	21 (40.4)	0.765
Taste–smell problem, *n* (%)	41 (50.0)	28 (53.8)	0.664
Duration of symptoms, days, mean (SD)	36.4 (6.4)	55.6 (12.1)	0.659
Number of symptoms, mean (SD)	4.7 (2.8)	5.0 (2.5)	0.595
Severe discomfort, *n* (%)	22 (26.8)	14 (26.9)	0.990
Hospitalization, *n* (%)	4 (4.9)	8 (15.4)	<0.05^**^
Duration of hospitalization in days, mean (SD)	0.3 (0.2)	2.0 (0.8)	<0.05^**^
Admission in ICU, *n* (%)	1 (1.2)	5 (9.6)	<0.05^**^
Duration of ICU admission in days, mean (SD)	0.1 (0.1)	1.5 (0.8)	<0.05^**^
**Post-COVID-19 neuropsychological assessment**
IADL partially autonomous, *n* (%)	4 (4.9)	2 (3.8)	0.778
BFI severe, *n* (%)	0 (0.0)	3 (6.4)	n/a
PHQ-9 mild to severe, *n* (%)	30 (36.6)	23 (44.2)	<0.05^**^
GAD-7 mild to severe, *n* (%)	26 (31.7)	19 (36.5)	0.564
Abnormal m-TICS, *n* (%)	15 (18.3)	11 (21.2)	0.896
Abnormal O-TMT-A, *n* (%)	6 (7.3)	6 (11.5)	0.404
Abnormal O-TMT-B, *n* (%)	8 (9.8)	4 (7.7)	0.668

SD, standard deviation; IQR, interquartile range; COPD, chronic pulmonary obstructive disorder; ICU, intensive care unit; GAD-7, generalized anxiety disorder-7; PHQ-9, patient health questionnaire-9; IADL, instrumental activities of daily living; BFI, brief fatigue index; TICS-m, modified telephone interview for cognitive status; O-TMT-A, oral trail making test A; O-TMT-B, oral trail making test B. A χ^2^ test was used to identify differences in proportions across categories. For normally distributed continuous data, a Student *t*-test was used to test differences between groups or by Mann–Whitney U-test for non-normally distributed data. Bold characters indicate significant differences (*p*≤0.05). *p* value <0.1 (^*^), *p* value < 0.05 (^**^), and *p* value< 0.01(^***^).

## 4. Discussion

The prevalence of cognitive sequelae post-COVID-19 infection has been quite unanimously reported in 50–67% of patients (2, 5–9, 16). However, cognitive profiling varies between studies. The discrepancies in the cognitive profiles in the literature can be explained by differences in study designs, which can dramatically influence the results. First, cognitive assessments were done as early as 3 weeks to as late as 12 months post-acute infection phase, making comparisons difficult between studies (2–9, 11–13, 15–17, 39, 42, 55, 56). Second, studies with cognitive assessment beyond the 12-month mark had heterogeneous populations, as one study focused exclusively on an elderly population (39), while others included only hospitalized patients (55, 56). Third, some studies utilized Montreal Cognitive Assessment (MoCA) as the only cognitive assessment tool (40, 41, 56), contrasting with other teams who did an extensive neuropsychological assessment in a population similar to ours, but with a higher rate of hospitalization (47%) (42). Although the MoCA has demonstrated its utility as a cognitive screener and is widely used in research studies, it suffers from several limits. Indeed, some studies have demonstrated a lack of sensitivity, a lack of robust correspondence between individual tests and their assumed cognitive domains, and low reliability in non-clinical populations. The MoCA should not be viewed as a substitute for more in-depth neuropsychological assessment when domain-specific information is required (57–59).

In our study, we used an extensive set of neuropsychological testing, focusing on executive tasks and psychological symptoms, and our result may be more applicable to the general population as a low percentage of our population had been hospitalized (9%). One recent longitudinal study followed older survivors of COVID-19 up to 1-year post-infection and reported temporal changes in the cognitive profile of patients, with a higher speed of cognitive decline associated with a more severe COVID-19 infection (39). Similarly, we found that cognitive inhibition deficits were associated with more severe COVID-19 infection as defined by hospital and ICU admissions and lengths of stay. Similarly, in our study, the cognitive profile of participants assessed before 6 months post-infection was different post-infection was different compared to the cognitive profile of participants assessed after 6 months post-infection. The prevalence of impaired cognitive efficiency decreased to reach 0% after 16 months post infection. Inversely, the prevalence of cognitive inhibition deficits remained unchanged and relatively high from 1 to 16 months post-infection. The profile of mild impairment varied between the early and late assessment groups, as nearly all patients who exhibited mild impairment after 6 months displayed an inhibition deficit. Those results suggest that patients with moderate impairment after COVID-19 infection might have a long-lasting impairment, while patients with mild cognitive impairment might see that most of their deficits resolve within the first 6 months post-infection, except for cognitive inhibition.

Although our small sample size may limit the interpretation of those findings, they support the existing literature linking the severity of infection to the frequency and severity of cognitive and neuropsychiatric impairments (39). Mattioli et al. found that intensive care admission predicted an inferior MMSE score (15), while ICU admission was associated with poorer executive function test results in another study by Almeria et al. (12). Furthermore, the number of days spent in the intensive care unit has been linked to inferior Stroop test interference suppression scores (60). In our study, in addition to the infection severity, no other sociodemographic or medical history variables were associated with cognitive inhibition or with cognitive efficiency or flexibility, which is consistent with previous studies (13, 17, 21). However, an abnormal TICS-m score was associated with anxiety and depression at the time of assessment, and an abnormal Hayling and Brixton score was associated with depression. This phenomenon can be explained by the literature on the suboptimal control of selective attention and working memory in patients with major depressive disorder. Functional MRI studies have demonstrated that participants with depression display significantly inferior dorsolateral prefrontal cortex activation during tasks involving working memory and executive functions than healthy controls (61). This area of the brain is highly correlated with working memory and attention control (62). Some data point toward the conclusion that these deficits in executive functions are in fact due to inhibition dysfunction and that, inversely, intact inhibition function is effective in decreasing the activation of irrelevant stimuli and their intrusion into the working memory, in addition to preventing prolonged rumination and poor emotional regulation (19). Thus, the prediction of an abnormal inhibitory response test in patients with symptoms of depression could be explained by dysfunctional prefrontal circuitry. Alternatively, the high prevalence of depression observed in post-COVID-19 syndrome could accentuate the frequency and severity of inhibition deficits through the mechanisms herein discussed. The orbitofrontal cortex-mediated deficits could perpetuate a vicious cycle.

A highlight of this study is the absence of an association between inhibition deficit and the delay since the initial infection, which may signify that beyond the acute phase and up to 16 months, these cognitive sequelae do not improve in the absence of treatment. Alternatively, it may suggest that inhibition deficits persist longer than other cognitive deficits or that inhibition deficit is more sensitive to COVID-19’s effects on the brain. While being a validated and normalized cognitive assessment to evaluate cognitive flexibility and inhibition (49), no other COVID-19 studies, to our knowledge, have evaluated frontal inhibition functions with the Hayling and Brixton test. Instead, studies used the Stroop Victoria test, which also assesses executive functions, with the sub-task of interference suppression specifically evaluating cognitive flexibility and inhibition (63). Similar to our findings, slower reaction time during interference suppression has been reported with the Stroop test in participants who have had COVID-19 when compared to healthy controls (21, 22), reflecting a poorer ability to restrain unsuitable or irrelevant responses (23). These functions entail having the capacity to regulate one’s attention, behavior, thoughts, and/or emotions in order to counteract pre-potent or inappropriate reactions (18). As such, deficits in inhibition due to lesions of the orbitofrontal cortex classically ensure catastrophic changes, marked by impulsivity and lack of judgment, leading to inappropriate social behaviors (62). In the case of post-COVID-19 syndrome, the consequences seem to be more subtle.

The absence of correlations between an abnormal Hayling and Brixton test and other neurocognitive tests, such as the TICS-m or O-TMT, suggests that inhibition deficits could be independent of other cognitive impairments in post-COVID-19 patients and share pathophysiological mechanisms with the SARS-CoV-2 infection. Due to the anatomical contiguity of the cribriform plate and the orbitofrontal cortex, direct viral invasion through the olfactory tract could explain why inhibition deficits are a marker of greater sensitivity than other cognitive impairments to evaluate COVID-19’s effects on the brain, while local neuronal damage could explain the persistence of the loss of function. Imaging studies support this hypothesis as orbitofrontal cortex atrophy and tissue damage to areas functionally connected to the primary olfactory cortex were observed in post-COVID-19 patients (64) Moreover, PET scan studies have revealed reduced glucose uptake in the orbitofrontal cortex and associated regions (65–67). Our findings support the hypothesis that inhibition deficit is an executive function that is highly vulnerable to the cognitive impact of the COVID-19 infection in comparison with other cognitive functions. Consequently, inhibition may be a more sensitive executive function for the long-term assessment of post-COVID-19 cognitive impairment and help reduce between-study variability in medium- to long-term cognitive impairment prevalence. While this finding should be validated by studies with larger sample sizes and further elucidated by robust research designs, this finding highlights the importance of not overlooking inhibition function in the clinical management of patients’ post-COVID-19 infection.

## 5. Limitations

Due to our cross-sectional study design and our small sample size, one should interpret the results with caution. Indeed, the authors acknowledge some limitations that are worth mentioning.

First, the length between acute COVID-19 infection and neuropsychological assessment was not evenly distributed during the study period, which might have increased both type I and type II errors in our comparative analyses between early and late assessment groups. Moreover, even if the population was randomly sampled, some significant differences were found between groups, regarding education level and preferred language, which could be confounders.

As our province is bilingual, we recruited both French- and English-speaking participants, portraying a more accurate sample of our regional population. However, although the Hayling and Brixton test is normed and validated for each language population, the scoring method differs in both versions and uses eight categories to describe the level of inhibition functioning in the English version and only five categories in the French version, which prevented us from stratifying our analyses by severity levels of inhibition. We, therefore, had to dichotomize the sample in normal versus abnormal inhibition to enable comparisons between language groups.

Furthermore, we must take into consideration the length of the interview which could have led to mental fatigue during neuropsychological testing, the global bilingualism of our population that could limit lexical access in a single language, and the pandemic’s social context as these variables may affect the interpretation of our data and may worsen the Hayling and Brixton test results. In addition, the high prevalence of fatigue and depression in our sample might have led to an overestimation of inhibition deficits as both may lead to sub-optimal response inhibition performance.

Our study population was infected between September 2020 and August 2021, signifying that the causal virus strain was primarily the Delta variant and that participants were not vaccinated. Thus, different outcomes could be expected with a different virus strain in the case of reinfection or in a vaccinated population.

The response rate of this survey (137/179 = 77%) might not reflect the general population.

Finally, the absence of a control group of individuals with no COVID-19 infection and the absence of cognitive profile information before COVID-19 infection, which means that this study might include patients who already have potential cognitive dysfunction, may limit the interpretation of our findings on the prevalence of cognitive impairment but is arguable as the goal of the study was to assess the dynamic cognitive profile changes in the infected population.

## 6. Conclusion

This study revealed an inhibition deficit in 38.8% of post-acute COVID-19 patients. A higher frequency and severity of impairment were correlated with the severity of depression, hospital admission, length of hospital stay, ICU admission, and length of ICU stay. While cognitive efficiency and mental flexibility improved with time, inhibition deficit was shown to be long-lasting in this investigation, and the length of time since the initial infection had no bearing on the severity of the impairment. Furthermore, the considerable prevalence of inhibition deficit in our patients reinforces the theory of direct CNS invasion of COVID-19 from the olfactory tract, due to the anatomical contiguity of the cribriform plate and the orbitofrontal cortex. Other neuropsychological scales and general health questionnaires support and validate previous evidence of cognitive impairment, neuropsychiatric illness, and fatigue following COVID-19.

In light of this study’s results and the existing literature, it is apparent that there is a need for increasing the availability of programs dedicated to physical and cognitive rehabilitation in post-COVID-19 syndrome patients. The variability in cognitive assessment between studies reflects different assessment tools, research designs, populations with different cognitive impairment risk factors, or simply different cognitive functions targeted by the studies. Based on our findings, we recommend that, upon replication and validation of our results, inhibition should be systematically assessed along global cognitive functions in future research to monitor the long-term course of such deficiencies, in addition to the evaluation of rehabilitation’s effect on cognitive and neuropsychological impairments.

## Data availability statement

The raw data supporting the conclusions of this article will be made available by the authors, without undue reservation.

## Ethics statement

The studies involving human participants were reviewed and approved by Vitalité Health Network’s Research Ethics Committee and Research Ethics Committee of Université de Moncton. The patients/participants provided their written informed consent to participate in this study.

## Author contributions

JS: data collection, interpretation of results, and redaction of the original manuscript. ZB: data collection and contribution to original manuscript redaction. CJ: statistical analysis, interpretation of results, and manuscript redaction. KC: database management and manuscript review. SC, MA-Q, and EL: data collection and manuscript review. M-CL: neuropsychological testing design. GG: infectiologist scientific study design contribution. JJ: study design, redaction of protocol, interpretation of results, and manuscript review. LC-W: study design, redaction of protocol, interpretation of results, and manuscript redaction. All authors contributed to the article and approved the submitted version.

## Funding

The authors disclose receipt of the following financial support for the research of this article from the Center de Formation Médicale du Nouveau-Brunswick (CFMNB), COVID-19 Strategic Grant, 2nd competition-2020.

## Conflict of interest

The authors declare that the research was conducted in the absence of any commercial or financial relationships that could be construed as a potential conflict of interest.

## Publisher’s note

All claims expressed in this article are solely those of the authors and do not necessarily represent those of their affiliated organizations, or those of the publisher, the editors and the reviewers. Any product that may be evaluated in this article, or claim that may be made by its manufacturer, is not guaranteed or endorsed by the publisher.
